# Evaluation of modified coblation endoscopic lingual lightening in multilevel surgery for obstructive sleep apnea hypopnea syndrome: an open intervention study

**DOI:** 10.1007/s11325-023-02912-2

**Published:** 2023-10-16

**Authors:** Xiangqiang Duan, Minhui Zhu, Caiyun Zhang, Meng Li, Yupeng Cai, Shicai Chen, Hongliang Zheng

**Affiliations:** 1 Department of Otorhinolaryngology Head and Neck Surgery, The First Affiliated Hospital of Naval Medical University, No. 168, Changhai Rd, Shanghai City, 200433 China; 2 Department of Medical Imaging, The First Affiliated Hospital of Naval Medical University, Shanghai City, China

**Keywords:** Obstructive sleep apnea, Retropalatal obstruction, Retrolingual obstruction, H-uvulopalatopharyngoplasty, Modified coblation endoscopic lingual lightening

## Abstract

**Purpose:**

To evaluate the efficacy and safety of modified coblation endoscopic lingual lightening to address retrolingual obstruction in multilevel surgery for obstructive sleep apneae (OSA).

**Methods:**

Patients with OSA due to retropalatal and retrolingual obstructions were enrolled. Group 1 consisted of patients who underwent modified coblation endoscopic lingual lightening combined with H-uvulopalatopharyngoplasty, while group 2 comprised patients treated by H-uvulopalatopharyngoplasty alone. Objective parameters and subjective evaluations were recorded preoperatively and at 6 months postoperatively.

**Results:**

The mean (standard deviation) apnea-hypopnea index (AHI) declined from 51.5 (18.9) to 14.3 (7.2) in group 1, and from 51.7 (15.8) to 28.5 (16.9) in group 2. The mean (standard deviation) percentage change in AHI was higher in group 1 than in group 2 (73.2 [10.9] vs. 48.9 [22.4], *P* < 0.01). The surgical response rate differed significantly between groups 1 and 2 (88.5 [23/26] vs. 46.7 [14/30], *P* < 0.01). Other outcomes, including the lowest oxygen saturation, Epworth Sleepiness Scale score, snoring visual analog scale score, and subjective improvement rate, were also significantly better in group 1 than in group 2.

**Conclusion:**

Without increasing complications, modified coblation endoscopic lingual lightening significantly improved surgical outcomes as part of multilevel surgery in patients with OSA due to multilevel obstruction.

## Introduction

Multilevel obstruction is a common occurrence in patients with obstructive sleep apnea (OSA), particularly in those with severe cases [1]. The retropalatal and retrolingual areas are the two most commonly obstructed sites [2–6]. Uvulopalatopharyngoplasty (UPPP) is the most frequently performed surgical procedure for the treatment of OSA related to retropalatal obstruction. In 2005, Han et al. introduced a revised version of UPPP known as H-UPPP [7]. This procedure has been shown to be effective in treating retropalatal obstruction with fewer complications than classic UPPP. However, H-UPPP does not address upper airway obstruction caused by tongue-related factors. Tongue-related upper airway obstruction typically presents in two distinct forms [8]. First, the tongue retracts and obstructs the retrolingual area by moving towards the posterior pharyngeal wall. Second, the tongue exerts pressure on the soft palate, leading to retropalatal obstruction by pushing it against the posterior pharyngeal wall. Thus the tongue not only causes retrolingual obstruction, but also retropalatal obstruction.

Due to its minimally invasive nature, the transoral approach has become the primary method for treating upper airway obstruction caused by tongue-related factors. The significance of the tongue base in pathogenesis of retrolingual obstruction has been widely acknowledged. A variety of transoral tongue base techniques have been devised to enlarge the retrolingual space, such as endoscopic coblator open tongue base resection, coblation endoscopic lingual lightening, submucosal minimally invasive lingual excision, endoscopic partial midline glossectomy, and the Robo-Cob technique [1, 8–16]. In these studies, surgical response rate was defined as a postoperative apnea/hypopnea index (AHI) of less than 20 events per hour and at least a 50% reduction in baseline AHI. The surgical response rates of these tongue base procedures exhibit significant variability, ranging from 56.3 to 78.7%, and are often associated with a higher incidence of postoperative complications, including bleeding, pain, infection, edema, lingual paralysis, and taste disturbance.

Most transoral approach procedures dealing with upper airway obstruction caused by tongue factors focus on the tongue base, and neglect the factors related to the tongue body [1, 8–16]. This focus on the tongue base is likely due to the concerns that surgery on the tongue body may compromise tongue function and increase postoperative complications. Additionally, the surgical site was left unsutured following treatment of the tongue [1, 8–16]. The exposure of unsutured surgical sites to oral fluids and food can heighten the likelihood of hemorrhage, infection, and discomfort. The aforementioned characteristics are typical of contemporary transoral procedures employed for the management of upper airway obstruction associated with tongue-related factors. Future research will focus on refining the current transoral tongue surgery techniques to enhance their effectiveness, minimize postoperative complications, and augment patient and sleep surgeon acceptance.

In contrast to previous procedures, our approach involves treating both the tongue base and tongue body together. Additionally, we close the operative area in the lingual region using interrupted sutures. This study sought to determine if the modified approach is more effective in improving surgical outcomes.

## Materials and methods

This study was approved by the Institutional Review Board of Changhai Hospital, which is affiliated with the Second Military Medical University. The study conforms to the Declaration of Helsinki. All patients signed an informed consent.

### Subjects

**Fig. 1:**
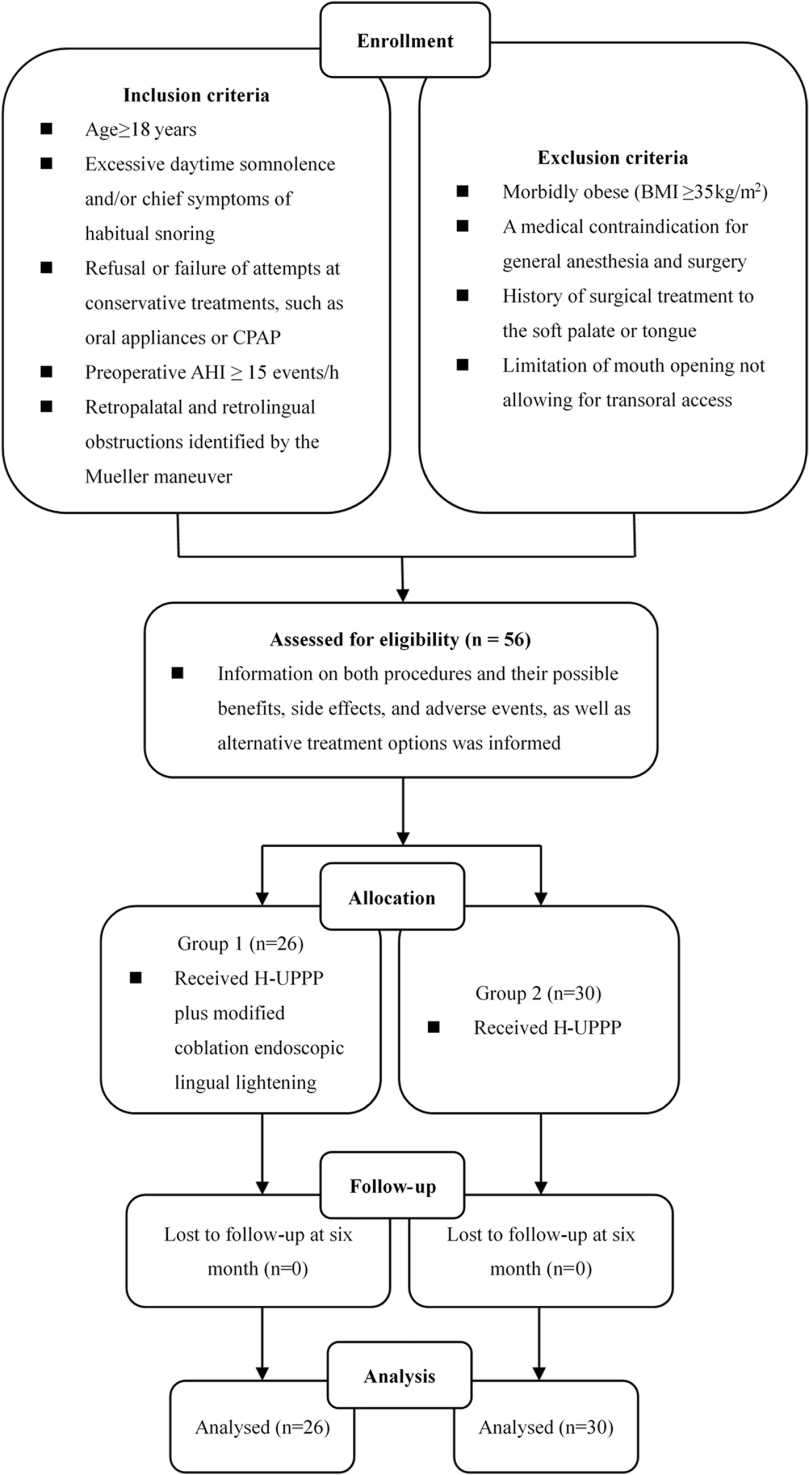
Flow diagram of the study

We enrolled patients with OSA who had retropalatal and retrolingual obstruction and were treated at the Department of Otorhinolaryngology Head and Neck Surgery, Changhai Hospital affiliated with the Second Military Medical University between August 2017 and April 2022. These patients either failed or declined continuous positive airway pressure (CPAP) therapy. All participants were above 18 years and had no history of upper airway surgical treatments. The diagnostic criteria for OSA were based on the 2012 guidelines of the American Academy of Sleep Medicine [17]. Anthropometric profiles and physical findings of the pharynx (including tonsil grade using Friedman stage, Friedman stage, modified Mallampti grade, and Fujita classification) were collected. The study’s flow diagram is depicted in Fig. 1.

Prior to surgery, detailed explanations were provided to the study participants regarding surgical options and associated risks for retropalatal and retrolingual obstruction. Some patients opted for group 1 to address both retropalatal and retrolingual obstructions simultaneously. However, other patients expressed concerns about potential surgical trauma and associated risks that may arise from concurrent treatment of both conditions. Therefore, they opted for group 2 to address only the retropalatal obstruction initially and then observe the surgical effect. If the outcome was unsatisfactory, they would consider a second surgery to tackle the retrolingual obstruction. Group 1 comprised patients consisting of moderate and severe cases of OSA who underwent modified coblation endoscopic lingual lightening combined with H-uvulopalatopharyngoplasty (H-UPPP). Group 2 consisted of patients with moderate to severe OSA who underwent H-UPPP alone. All patients provided informed consent, underwent their selected surgeries, and were included in the final analysis. We collected both subjective and objective measures of effectiveness while also documenting complications that arose.

Effectiveness was assessed through a comparison of subjective and objective parameters before and after surgery. Subjective evaluations included daytime sleepiness, as measured by the Epworth Sleepiness Scale (ESS), and snoring, which was evaluated by bed partners using a visual analog scale (VAS) [1, 8, 10]. The objective outcome measures encompassed polysomnographic parameters and endoscopic data. All complications were meticulously documented. A successful surgical outcome was defined as a postoperative AHI of less than 20 events per hour and at least a 50% reduction in the baseline AHI [8, 10, 11, 13, 18, 19].

### Endoscopy

Our endoscopic approach was consistent with that described by Askar et al. [20]. Patients underwent awake endoscopic examinations (Olympus, Tokyo, Japan) at 1 week prior to and 6 months following surgery. Both nasal cavities were sprayed twice with 1% (w/v) ephedrine and 1% (w/v) dicaine. Subsequently, all patients were positioned in a supine posture without any shoulder pads, and electronic nasolaryngoscopy fibers were introduced into the nasal cavity to conduct a comprehensive examination and capture photographic images of the entire upper airway. The Müller’s maneuver (MM) was performed with the patient’s nose pinched, mouth closed, and deep breathing. Obstruction was defined as a ≥ 50% reduction in cross-sectional area at any level.

### Surgical procedures

All patients underwent CPAP treatment for 1 week prior to surgery in order to correct systemic hypoxia and enhance surgical tolerance [21–23]. All surgical procedures were performed under general anesthesia administered via nasotracheal intubation. All patients were positioned in a supine position with their necks immobilized and heads extended. Optimal exposure was ensured using a standard mouth retractor and an appropriate tongue blade. The Coblator II Surgery System and EVac 70 coblation wand (ArthroCare Corporation, Sunnydale, CA, USA) were employed, with coblation settings of 7 in ablation mode and 3 in coagulation mode.

In group 1, the procedure of H-UPPP was conducted as a primary step, followed by modified coblation endoscopic lingual lightening. In contrast, only H-UPPP was performed in group 2.

#### H-UPPP

**Fig. 2:**
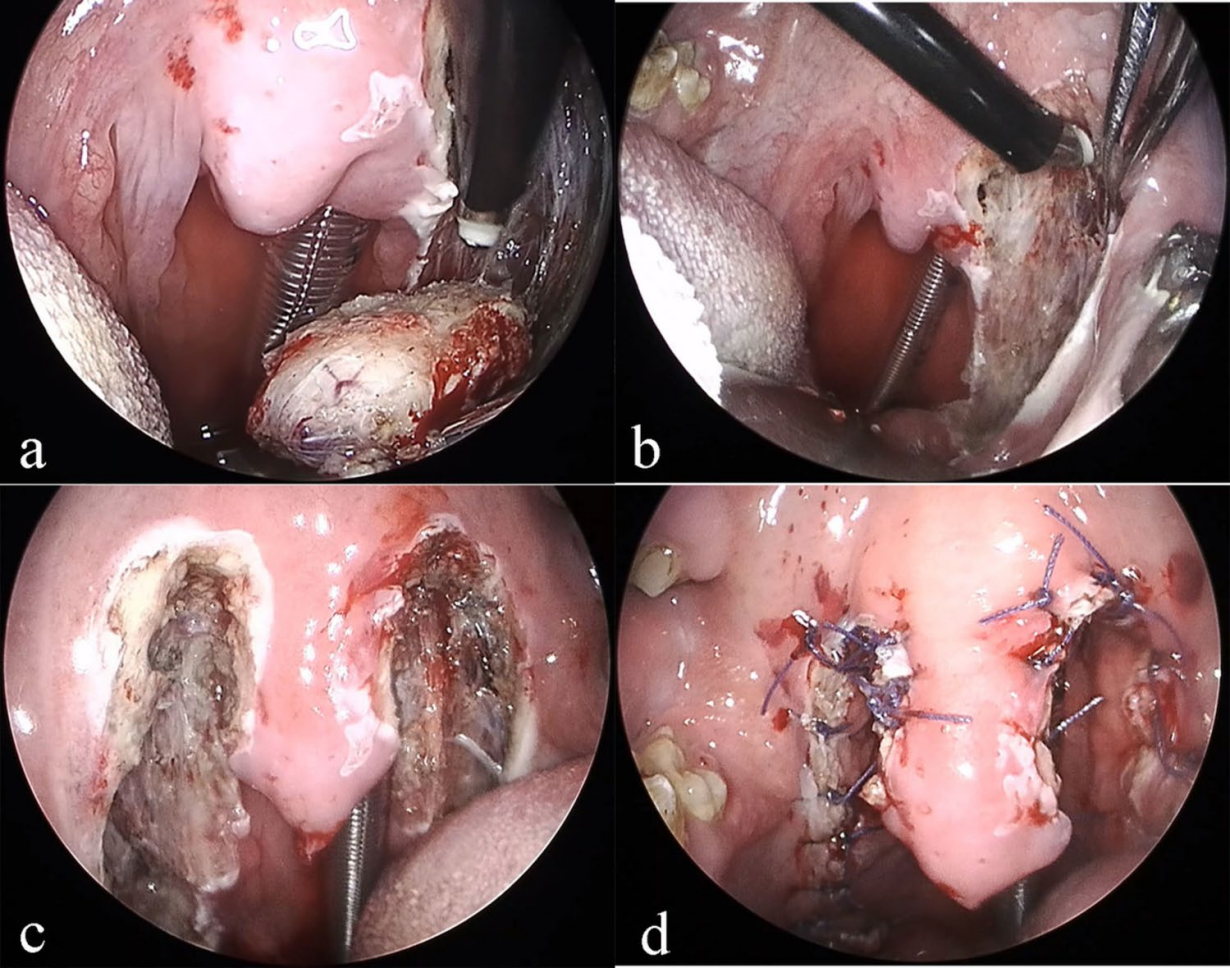
The procedural steps of H-UPPP surgery (**a**–**d**). First, tonsillectomy was performed (**a**), followed by ablation of redundant pharyngeal mucosa and submucosal tissue (**b**). Then, two inverted V-shaped incisions were designed along both sides of the uvula on the ventral surface of the soft palate (**c**). An overall manifestation of H-UPPP surgery is shown in **d**

A Davis mouth gag was utilized to facilitate oral access for the purpose of performing a bilateral tonsillectomy. Redundant pharyngeal mucosa and submucosal tissue were ablated to widen the oropharyngeal lumen. Two V-shaped incisions were made on both sides of the uvula, located on the ventral surface of the soft palate. Excess submucosal adipose tissue hidden in the spatium veli palatini was ablated. The margins of the musculus uvulae, levator palatine, and tensor palatini as well as their corresponding mucosal membranes were preserved. The ventral and dorsal margins of the preserved uvula mucosal membrane were sutured with interrupted stitches to reconstruct the uvula. The palatopharyngeal and palatoglossal arches were secured with interrupted sutures to stabilize and enlarge the oropharyngeal airway. The process for H-UPPP is depicted in Fig. 2.

#### Modified coblation endoscopic lingual lightening

**Fig. 3:**
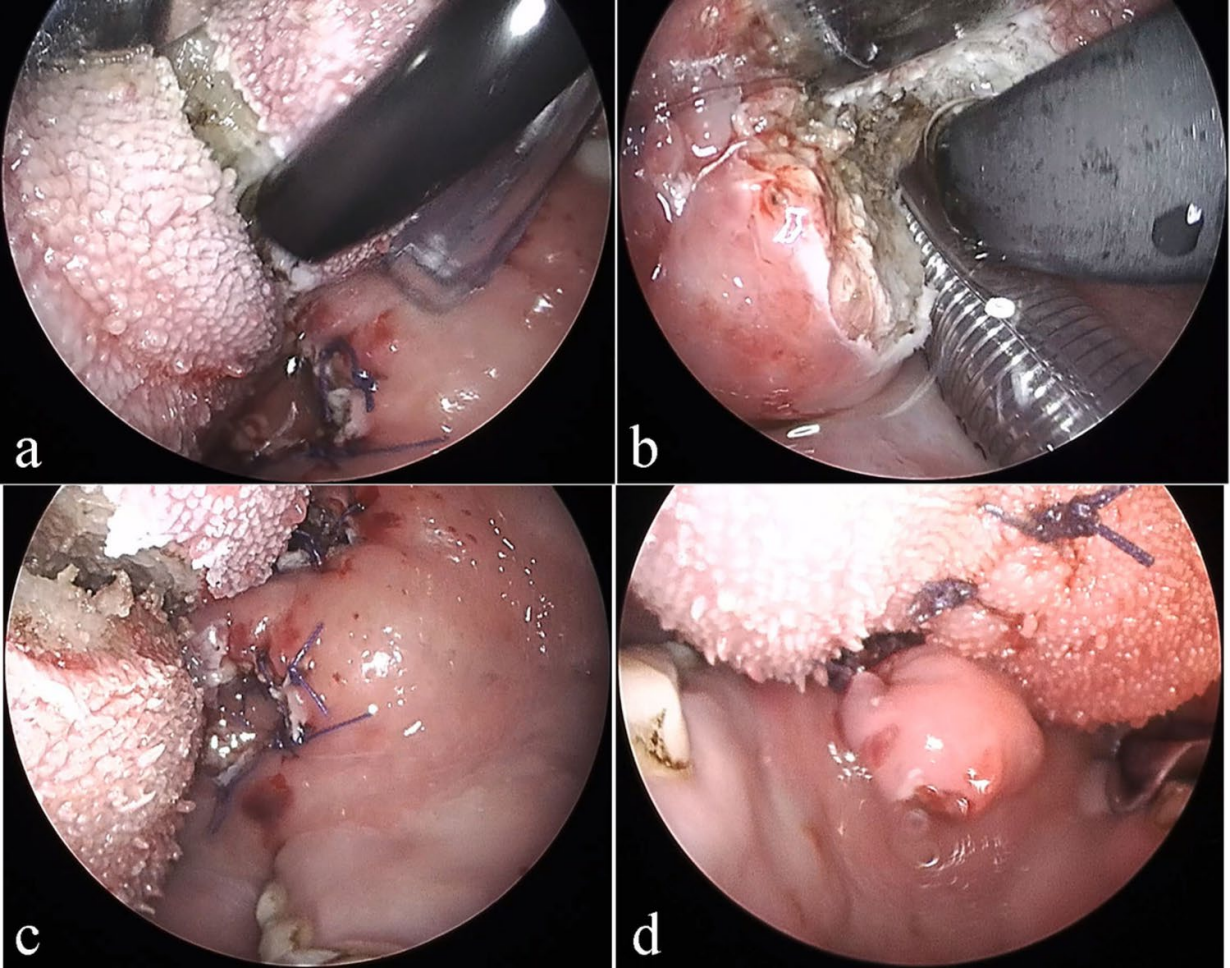
The procedural steps of modified coblation endoscopic lingual lightening procedure (**a**–**d**). Panel **a** shows ablation of the tongue body, while panel **b** displays ablation of the tongue base. The overall appearance of modified coblation lingual lightening before and after suture are shown in panels **c** and **d** respectively

A 2–0 silk suture was secured to the tip of the tongue for retraction, and the tongue was protracted towards the chest to augment surgical access in the retrolingual region. A side mouth gag was positioned in the molar region to achieve mouth opening, and an appropriate tongue blade was utilized to depress the dorsum of the tongue for optimal exposure of the surgical field. The suitability of different lengths of tongue blades was assessed until an optimal surgical view was achieved. A 70° rigid sinus endoscope (Karl Storz, Tuttlingen, Germany) was inserted transorally and positioned in an upward-facing orientation to provide visualization of the surgical field. The surgeon positioned themselves at the head of the bed, while the video system was strategically placed at the foot end. The coblator wand was gently bent for easy access to the surgical area. Lingual ablation was initiated 3–4 cm anterior to the circumvallate papillae and continued posteriorly along the midline until reaching the vallecular. The ablated area measured 2 cm in width and 1.5 cm in depth. After achieving complete hemostasis with the coblator, the lingual ablation site was closed using interrupted sutures with 2–0 absorbable suture. The procedure is illustrated in Fig. 3. The lingual ablation site was closed using interrupted sutures with 2–0 absorbable suture.

### Image analysis

**Fig. 4:**
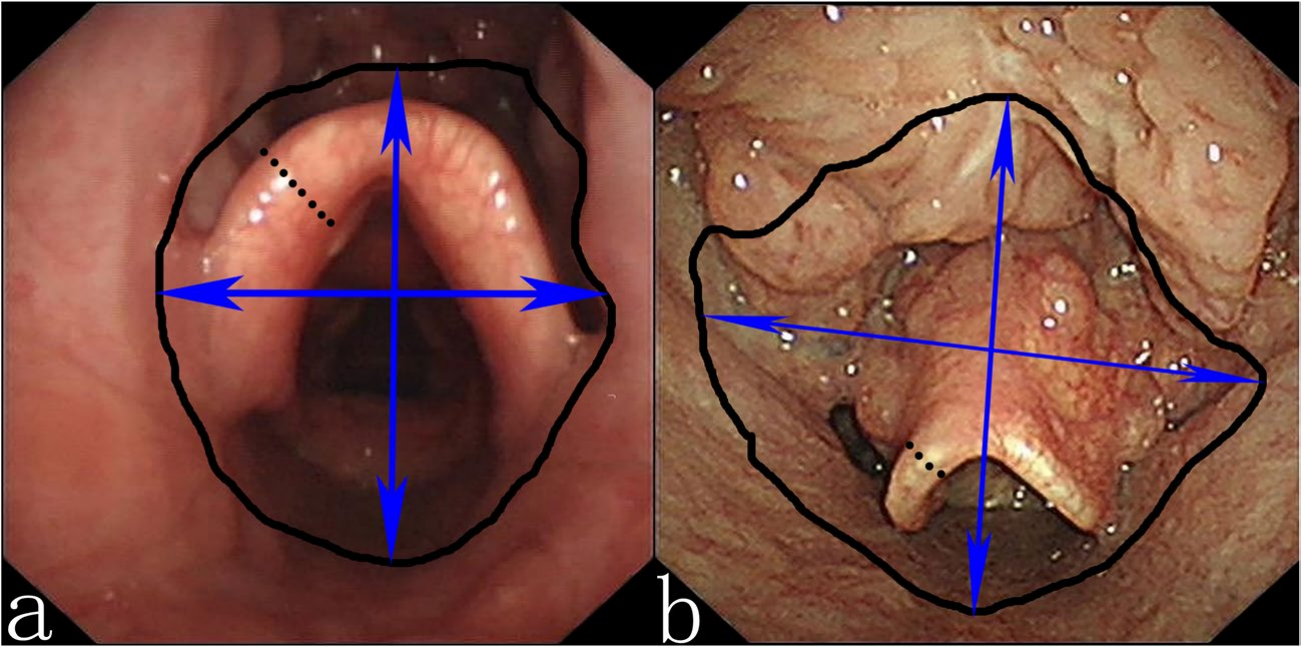
Retrolingual regions of group 1 pre- and post-surgery. During the Muller maneuver, a specific point of the epiglottis (indicated by a black dotted line) was measured both before (**a**) and after (**b**) surgery. The dimensions of this region were *X* pixels prior to surgery and *Y* pixels following the procedure. The scaling factor was expressed as the ratio of *X* to *Y*. The black continuous line represents the narrowest retrolingual plane, while the two double-headed blue arrows indicate its anteroposterior and transverse diameters

The endoscopically narrowest area in the retrolingual region was evaluated pre- and post-operatively. The employed image analysis methodology is consistent with that of Borek et al. [24]. The images captured during MM were analyzed using ImageJ software (https://imagej.net/, ImageJ (RRID: SCR_003070)). The pixel served as the fundamental unit of measurement for evaluating pre- and postoperative characteristics of the retrolingual region. The focal depth of retrolingual region may vary between preoperative and postoperative images of the same patient, resulting in a different standard pixel sizes for identical anatomical structures. Therefore, a scaling factor was employed to rectify this discrepancy. We measured a common anatomical distance of the epiglottis pre- and post-operatively. The scaling factor is calculated as the ratio of preoperative to postoperative epiglottic measurements. The corrected postoperative measurements were created multiplying the original measurements by this scaling factor. The percentage change was determined by subtracting the preoperative value from the corrected postoperative value, dividing the result by the preoperative value, and then multiplying it by 100%. See Fig. 4 for details.

### Postoperative care

Postoperatively, all patients were closely monitored in the surgical intensive care unit for a duration of 8 h. A prophylactic regimen of cefuroxime and dexamethasone sodium phosphate was prescribed for all patients over a 3-day period. Each patient received enteral nutrition via a nasogastric tube in the first postoperative week and was transitioned to an oral soft diet during the second postoperative week; thereafter, a normal diet was resumed. No precautionary tracheostomy was performed.

### Statistical methods

All statistical analyses were performed using SPSS software (https://www.ibm.com/products/spss-statistics, IBM SPSS Statistics (RRID: SCR_019096)). Continuous data were presented as means (standard deviations). The *t*-test was utilized for comparing normally distributed data with homogeneity of variance, while the Mann-Whitney *U* test was employed for comparing non-normally distributed data. The *χ*^2^ test was employed to analyze categorical variables. The statistical significance was determined by a *P*-value < 0.05.

## Results

### Patient characteristics

The demographic data of the two groups is shown in Table 1. All participants in our recruited groups were diagnosed with retropalatal and retrolingual obstruction, leading to the classification of all patients in both groups as type 2 according to the Fujita classification. There were no significant differences in preoperative age, gender distribution, BMI, tonsil size, Friedman stage, modified Mallampati classification, AHI, lowest oxygen saturation, ESS score, and snoring VAS score between the two groups with a *P*-value greater than 0.05.

**Table 1:** Baseline characteristics of the two groups

Variable	Group 1 (*n* = 26)	Group 2 (*n* = 30)	*P*
Age, mean (SD), year	46.1 (8.7)	44.8 (10.2)	0.61
Male–female ratio	24:2	29:1	0.90
BMI, mean (SD), kg/m^2^	28.5 (3.4)	28.3 (3.8)	0.84
Tonsil grade	1.8(0.7)	1.5(0.6)	0.12
Friedman stage	2.6(0.5)	2.7(0.6)	0.58
Modified Mallampati grade	2.3(0.6)	2.2(0.5)	0.57
AHI, mean (SD), events/h	51.5 (17.9)	51.7 (15.8)	0.96
Lowest oxygen saturation, mean (SD), %	67.3 (10.1)	67.8 (8.2)	0.83
ESS score, mean (SD)	14.7 (2.2)	14.3 (2.4)	0.55
Snoring VAS, mean (SD)	8.2 (1.1)	8.5 (1.0)	0.23

Group 1 represents modified coblation endoscopic lingual lightening plus H-UPPP; Group 2 represents H-UPPP only. *SD* standard deviation, *BMI* body mass index, *AHI* apnea hypopnea index, *ESS* Epworth Sleepiness Scale, *VAS* Visual Analog Scale

Table 2 illustrates the preoperative and postoperative changes in BMI, AHI, lowest oxygen saturation, ESS score, and snoring VAS score for both group 1 and group 2. With the exception of BMI, all parameters exhibited significant differences before and after surgery (*P* < 0.05).

**Table 2:** Comparison of pre- and postoperative outcomes between groups 1 and 2

Variable	Mean (SD)				*P*
	Preoperative	Postoperative	Change	Change (%)	
Group l					
AHI, events/h	51.5 (18.9)	14.3 (7.2)	− 37.2 (13.0)	− 73.2 (10.9)	< 0.01
Lowest oxygen saturation, %	67.3 (10.1)	83.7 (6.5)	16.4 (8.6)	26.6 (18.0)	< 0.01
ESS score	14.7 (2.2)	5.2 (2.3)	− 9.5 (1.6)	− 65.6 (12.8)	< 0.01
Snoring VAS score	8.2 (1.1)	3.4 (1.4)	− 4.8 (1.2)	− 58.8 (15.3)	< 0.01
BMI	28.5 (3.4)	28.1 (2.7)	− 0.4 (1.4)	− 1.2 (4.7)	0.12
Group 2					
AHI, events/h	51.7 (15.8)	28.5 (16.9)	− 23.2 (9.1)	− 48.9 (22.4)	< 0.01
Lowest oxygen saturation, %	67.8 (8.2)	78.0 (7.3)	10.2 (6.7)	15.9 (11.1)	< 0.01
ESS score	14.3 (2.4)	7.6 (2.3)	− 6.7 (2.3)	− 46.9 (14.6)	< 0.01
Snoring VAS score	8.5 (1.0)	4.9 (2.1)	− 3.6 (1.9)	− 43.0 (23.6)	< 0.01
BMI	28.3 (3.8)	28.0 (3.3)	− 0.4 (1.3)	− 0.9 (4.7)	0.14

Group 1 represents modified coblation endoscopic lingual lightening plus H-UPPP; group 2 represents H-UPPP. *SD* standard deviation, *BMI* body mass index, *AHI* apnea hypopnea index, *ESS* Epworth Sleepiness Scale, *VAS* Visual Analog Scale

Significant postoperative increases were observed in the anteroposterior and transverse diameters, as well as the cross-sectional area of the narrowest retrolingual region in group 1. These findings were confirmed by endoscopic examination (Fig. 4 and Table 3).

**Table 3:** Endoscopic percent changes in narrowest area of retrolingual region during Muller maneuver before and after surgery in group 1

Measurement	% change	*P*^1^
Anteroposterior diameter	65.5 ± 40.8	< 0.01
Transverse diameter	67.6 ± 60.9	< 0.01
Cross-sectional area	88.4 ± 56.4	< 0.01

Group 1 represents modified coblation endoscopic lingual lightening plus H-UPPP

^1^Comparison before and after surgery

The postoperative results of the two groups are compared in Table 4. The mean AHI, ESS score, and snoring VAS score were significantly lower in group 1 than in group 2 (*P* < 0.01). The lowest oxygen saturation during sleep was significantly higher in group 1 than in group 2 (*P* < 0.01). The surgical response rate was significantly higher in group 1 than in group 2 (*P* < 0.01). Most patients with OSA initially seek treatment not only due to the negative effect on their health, but also because their bed partners report disruptive snoring. Therefore, subjective parameters were incorporated into the postoperative assessments. A reduction of ≥ 50% in both the self-reported ESS score and snoring VAS score as reported by the bed partner was considered indicative of subjective improvement. The subjective improvement rate reported by participants was significantly higher in group 1 compared to Group 2 (*P* < 0.01).

**Table 4:** Postoperative parameters of the two groups

Variable	Group 1 (*n* = 26)	Group 2 (*n* = 29)	*P*
BMI, mean (SD), kg/m^2^	28.1 (2.7)	28.0 (3.3)	0.875
AHI, mean (SD), events/h	14.3 (7.2)	28.5 (16.9)	< 0.01
Lowest oxygen saturation, mean (SD), %	83.7 (6.5)	78.0 (7.3)	< 0.01
ESS score, mean (SD)	5.2 (2.3)	7.6 (2.3)	< 0.01
Snoring VAS, mean (SD)	3.4 (1.4)	4.9 (2.1)	< 0.01
Surgical response rate, %	88.5 (23/26)	46.7 (14/30)	< 0.01
subjective improvement rate, %	76.9 (20/26)	40.0 (12/30)	< 0.01

Group 1 represents modified coblation endoscopic lingual lightening plus H-UPPP; group 2 represents H-UPPP. *SD* standard deviation, *BMI* body mass index, *AHI* apnea hypopnea index, *ESS* Epworth Sleepiness Scale, *VAS* Visual Analog Scale

### Complications

No significant complications were encountered during or after the operations. Most patients in both groups experienced transient velopharyngeal insufficiency, lasting from 5 days to 2 weeks, when attempting to swallow food or liquid while speaking or drinking rapidly. In group 1, there was only one instance of postoperative tongue bleeding which was effectively managed through the application of local pressure and injection of saline-diluted epinephrine. There were no cases of taste disturbance, dysphagia, or hypoglossal nerve paralysis.

## Discussion

Precise identification of the obstruction site in patients with OSA is an important initial step towards achieving successful surgical outcomes [25]. Awake endoscopy with MM and drug-induced sleep endoscopy (DISE) both provide a topographical evaluation of the upper airway. Both maneuvers help sleep surgeons in evaluating the level and the pattern of upper airway collapse. Currently, awake endoscopy with MM and DISE have become the most reliable tools in evaluating upper airway collapsibility of patients with OSA. However, both methods have their inherent limitations [26–28]. MM is an outpatient procedure that can be performed without drug induction. MM is less burdensome on the patient and hospital. However, controversy exists regarding the accuracy of evaluating upper airway collapsibility grade and pattern during MM due to its performance on awake patients. Due to its performance during sleep, DISE is widely regarded by sleep surgeons as the most dependable tool for assessing upper airway collapsibility. However, DISE has its limitations. DISE requires trained personnel, specialized facilities, and special rooms for the procedure. Additionally, the drug-induced sleep achieved during DISE only captures a limited portion of the complete sleep cycle, potentially failing to reflect all changes in upper airway morphology throughout the entire cycle. Furthermore, sleep surgeons encounter challenges in conducting follow-up assessments of postoperative patients using DISE due to its high cost and the requirement for general anesthesia. Consequently, DISE is not extensively utilized, particularly in low-income countries with restricted healthcare budgets.

Previous research has compared the outcomes of DISE and MM, revealing that both tests can significantly affect surgical planning and decision-making by providing precise assessments of upper airway collapse patterns and levels [3–5, 24–29]. Recent findings indicate that the obstruction pattern observed in DISE and MM is comparable, with the degree of collapse being the distinguishing factor [20, 30, 31]. Askar et al. demonstrated that positional awake endoscopy is a cost-effective and convenient outpatient procedure that yields comparable results to DISE in terms of upper airway collapse patterns and grades at all levels [20]. Unlike DISE, supine MM does not necessitate specialized anesthesia precautions, equipment, or facilities. The follow-up of patients utilizing supine MM is significantly simplified, and the issue of patient consent can be more readily resolved. Overall, the supine MM can provide valuable surgical insights into the level, pattern, and degree of upper airway collapse in patients with OSA. Therefore, we consider the supine MM to be a reliable tool for evaluating the three-dimensional anatomical topography of the upper airway and for making informed surgical plans and decisions.

The retrolingual obstruction is often observed in patients with severe OSA [4–6]. In order to minimize trauma, the transoral approach is typically preferred when treating such patients. Alternative tools include laser treatment, radiofrequency, and transoral robotic surgery. In recent years, otolaryngologists have increasingly utilized the coblation technique, which represents a cutting-edge and innovative approach to surgical procedures. Energized electrodes immersed in saline solution generate a plasma layer consisting of highly ionized particles, which effectively disrupt intercellular bonds within tissue and can be removed at low temperatures. Compared to other approaches, coblation is associated with reduced morbidity and fewer complications.

In 2006, Maturo and Mair first performed tongue base resection via coblation [32]. Li et al. pioneered coblation endoscopic lingual lightening for patients with OSA due to retrolingual obstructions [11]. The central portion of a hypertrophic tongue base is transorally ablated using coblation under endoscopic guidance. Bahgat et al. have introduced a novel transoral tongue base surgery technique, referred to as the “Robo-Cob” technique [9]. The exposure and operative technique are analogous to those of transoral robotic surgery. However, coblation is utilized for tongue base tissue resection instead of ablation. Additional coblation techniques have also been delineated [10, 14, 33, 34], all of which are viable and moderately effective in addressing retrolingual obstructions. However, the surgical response rates were suboptimal and postoperative morbidity and complications, including pain and bleeding, were relatively prevalent.

Previous treatments for retrolingual obstruction have primarily focused on the tongue base, neglecting the importance of the tongue body [35]. However, it is important to note that both the tongue base and body can contribute to not only retrolingual obstruction but also retropalatal obstruction [6, 36, 37]. Both regions are capable of causing retrolingual obstruction by moving against the posterior pharyngeal wall, and can also induce retropalatal obstruction by pushing the soft palate back against it. In clinical practice, the majority of patients with severe OSA exhibit hypertrophy of the tongue body, making treatment of this area a crucial aspect in managing their condition. The hypoglossal neurovascular bundle is situated at a depth greater than 1.5 cm and blood supply to the middle region of the tongue body is relatively poor [38]. Therefore, the surgical scope of tongue base and tongue body in our study was deemed safe. However, there is no standardized approach for managing the tongue body during treatment of retrolingual obstruction, and it remains unclear how much tissue can be safely resected to achieve optimal postoperative outcomes while minimizing functional impairment. In the current study, the ablation of the tongue body commenced 3–4 cm anterior to the circumvallate papillae and subsequently extended posteriorly along the midline towards the base of the tongue. The ablated region measured 2 cm in width and 1.5 cm in depth. Endoscopy findings revealed a significant increase in the anteroposterior diameter, transverse diameters, and cross-sectional area of the retrolingual region postoperatively.

The surgical response rates of these tongue base procedures exhibit significant variability, ranging from 56.3 to 78.7% [1, 8, 10, 11, 13, 39]. The current study demonstrated significant improvement in surgical response rates compared to previous research. Furthermore, at the 6-month follow-up, no lingual dysfunction was reported, indicating that our lingual surgery appears to be both safe and effective.

Previous research has suggested that the inclusion of lingual surgery in pharyngoplasty procedures may increase the likelihood of morbidity and complications, such as bleeding, pain, infection, edema, lingual paralysis, and taste disturbance [10, 11, 13, 18]. However, our own experience did not support this conclusion. The lingual ablated area was left unsutured in previous studies, whereas we opted to close the same using 2–0 absorbable threads. Lee et al. conducted a meta-analysis of nine studies on the use of plasma ablation for tongue base reduction in patients with OSA, revealing a 7.5% incidence rate of postoperative bleeding [12]. Sutures were not conduced in previous tongue base studies. In the current study, only one case of postoperative bleeding was encountered, indicating a low likelihood of such an occurrence. The reasons are twofold. First, suturing can occlude and ligate damaged blood vessels. Second, the ablated region lacking sutures induces the formation of protective pseudo-membranes that envelop the wound site and gradually dissolve during the process of healing. Premature displacement of these membranes and hemorrhage may occur due to early consumption of solid or hot foods, wound infection, and frequent coughing caused by abnormal throat sensations. Therefore, sutures can close the surgical wound, accelerate healing, and reduce postoperative bleeding.

There are several limitations to the study that need to be addressed. First, DISE was not utilized for the evaluation of the upper airway. Second, the efficacy of modified tongue surgery in multilevel surgery may be affected by the concurrent effect of H-UPPP. Therefore further investigation is required with further study. Third, the limited sample size utilized in this study restricted the extent of analysis regarding factors associated with outcomes. Additionally, a more extended follow-up period would facilitate the identification of ideal candidates for modified coblation endoscopic lingual lightening treatment.

## Conclusion

The findings of the current study suggest that the modified coblation endoscopic lingual lightening technique may be an effective treatment for OSA caused by retrolingual obstruction. The reduction of both the tongue body and base, with intermittent sutures used to close the ablated area on the tongue, appears to ensure safety and feasibility. 

## Data Availability

The datasets generated during the current study are not publicly available but are available from the corresponding author on reasonable request.
